# Isolated hypothalamic-pituitary langerhans’ cell histiocytosis in female adult: A case report

**DOI:** 10.1097/MD.0000000000013853

**Published:** 2019-01-11

**Authors:** Huiwen Tan, Kai Yu, Yerong Yu, Zhengmei An, Jianwei Li

**Affiliations:** a Department of Endocrinology and Metabolism; b Centre of Pituitary Adenoma and Related Diseases, West China Hospital, Sichuan University, Chengdu, China.

**Keywords:** anterior pituitary hormone deficiency (APD), central diabetes insipidus, hypothalamic-hypophysial axial mass, langerhans’ cell histiocytosis (LCH), multi-diciplinary treatment (MDT)

## Abstract

**Rationale:**

Langerhans cell histiocytosis (LCH) is characterized by clonal proliferation of immature dendritic cells, mainly affects children. LCH in adult sellar region is extremely rare. In literature, optimal treatments remain unclear and only a few cases of LCH were treated using surgery. Here, we present a rare case of isolated hypothalamic-pituitary LHC in female adult. We focused on elucidating the clinical manifestations and immunohistochemical features of LCH, and exploring the proper treatment in adults.

**Patient concerns:**

A 50-year-old woman was admitted to our hospital, presenting with polydipsia and polyuria for over 3 months.

**Diagnoses:**

Radiological studies revealed lesions (0.5 × 0.9 × 0.4 cm) on posterior pituitary and enlarged pituitary stalk, which was moderately enhanced on contrast magnetic resonance imaging (MRI) of sellar region. In biopsy, pathological examination of Langerhans cells were observed with positive S-100 protein and Ki-67 antigen markers, findings were sufficient to establish a diagnosis of central nervous system (CNS) LCH.

**Interventions:**

The patient with LCH restricted in the sellar region received both surgery and chemotherapy. Gamma knife radiosurgery was performed after diagnosed as central diabetes insipidus (CDI) induced by pituitary lesion. And tumorectomy was performed 5 years later. However, in the latest MRI in 2017, the nodular shadow became larger (about 1.4 cm), chemotherapy and further systemic therapy were given.

**Outcomes:**

At 12-month follow-up, no local reoccurrence was noticed.

**Lessons:**

For LCH, though difficult to be diagnosed and none defined standard therapeutic approach to adults, surgery should be considered if there are neurological symptoms or histological diagnosis. The present study showed that some manifestations can be meaningful when central nervous system (CNS) is involved. For complex diseases in the sellar region, multi-disciplinary team (MDT) model of diagnosis and treatment should be helpful for better clinical efficacy.

## 1 Introduction

Langerhans’ cell Histiocytosis (LCH) is a rare and multi-organ involved disease with unknown causes. It has been referred in the literature as Histiocytosis X, Letterer-Siwe disease, Hand-Schuller-Christian syndrome, eosinophilic granuloma of bone, self-healing reticulohistiocytosis, and more recently, Langerhans’ cell histiocytosis.^[1–2]^ LCH can affect bones, skins, lungs, and any other organs,^[3–6]^ and it may involve a single organ or system, or involve multiple organs or systems. Clonal proliferative disorder via BRAF V600E mutation, *MAP2K1* mutation, or activation of MAPK/ERK has been considered as possible pathogenesis for LCH.^[7–9]^ Furthermore, LCH shares characteristics of children dominance and variable severity. The manifestations of LCH are diverse, LCH patients may develop manifestations including painful bone, skin ulcer, polyuria, and et al, related to specific organ involvement.^[9]^ Thus, depending on the extent and site of the disease, treatments range from observation alone to systematic medication.^[10–11]^ However, evidence-based recommendations for adult LCH treatment are still lacking. In our case report, we tried to provide the proper management process to diagnose and treat adult LCH.

## 2 Case presentation

### 2.1 Ethical review and patient consent

The present study is dealt with the patient's medical records and related images and pathology reports. This observational study was approved by the Ethics Committee of West China Hospital of Sichuan University. And the clinical research was implemented according to the principles expressed in the World Medical Association Declaration of Helsinki and the International Ethical Guidelines for Biomedical Research Involving Subjects (GIOMS, Geneva, 1993). Written informed consent was given from the patient on each occasion of diagnostic examinations and therapeutic procedures and also for the publication of this case report.

### 2.2 Case report

**Figure 1:**
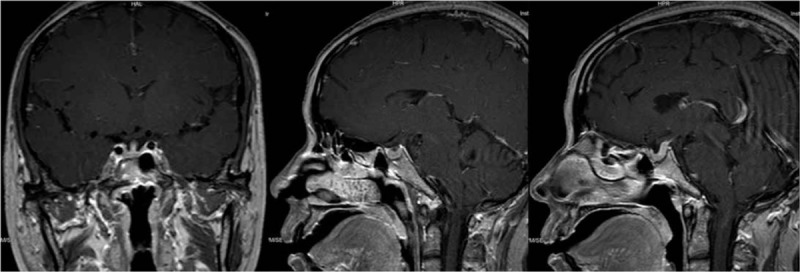
Enhanced MRI of sellar region in 2009. MRI before radiosurgery showed T1 low signal nodular intensity (0.5 × 0.9 × 0.4 cm) with homogeneous nodular enhancement of posterior pituitary, and also presented an enlarged pituitary stalk.

A 49-year-old woman complaining of polydipsia and polyuria for over 3 months, came to West China Hospital, Sichuan University (Chengdu, China) for further examination on November 6, 2009. Her family history was unremarkable and she did not have a history of traumatic injury or surgery on brain before admission. Physical examination was all normal. The results of biochemistry examinations are shown in Table 1A. Hormonal measurements showed elevated prolactin (PRL) level, but normal function of hypophysial-thyroid axis(HTA), hypophysial-adrenal axis (HPA) and hypophysial-gonadal axis (HGA) (Table 1B). Enhanced MRI of sella region showed lesions (0.5 × 0.9 × 0.4 cm) on posterior pituitary and enlarged pituitary stalk, but anterior pituitary, supra sella cistern, and adjacent skull were all normal on the MRI image (Fig. 1). Gamma knife radiosurgery was performed after diagnosed as central diabetes insipidus (CDI) induced by pituitary lesion.

**Table 1:**
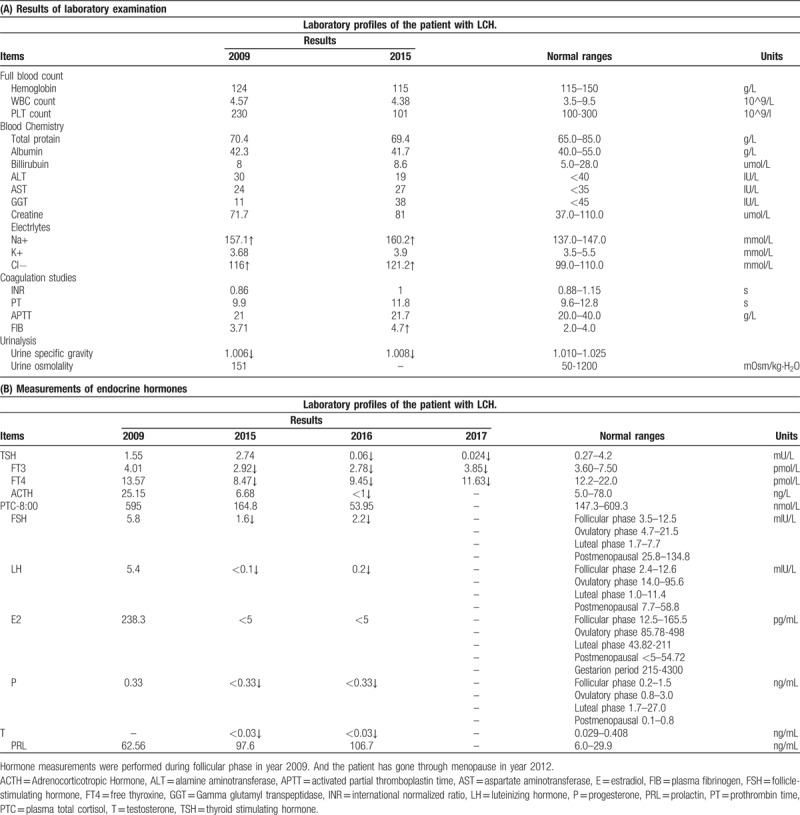
Laboratory profiles of the patient with LCH.

**Figure 2:**
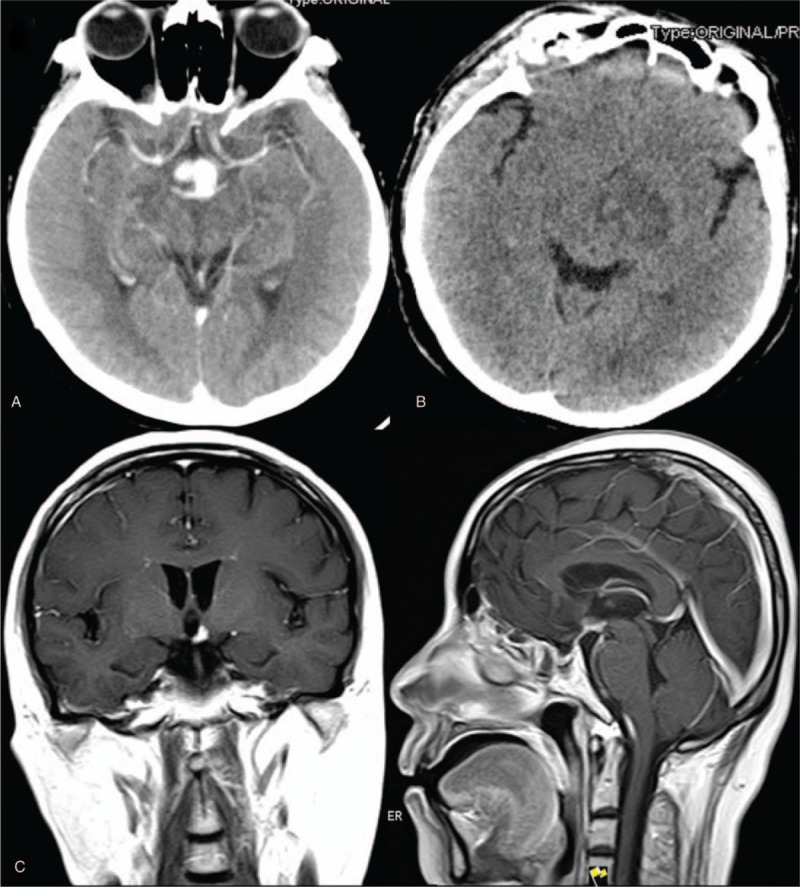
Head CT and MRI in 2015. A) Pre-operation CT showed soft tissue density shadow (1.6 × 1.3 cm) with irregular shape and well-defined margin in the sella region, and showed homogeneous enhancement. But encephalocoele, cisterns and skull bones had no abnormal signals on CT image. B) Post-operation CT showed the bone flap of the right frontal bone with scalp soft tissue swelling and accumulation of gas in surgical area. A mixture of air, edematous fluid, and hemorrhage were seen under the bone flap. Right frontal lobe was slightly compressed. There was intracranial air, thin layer of subdural edematous fluid in bilateral frontotemporal area and left side of falx cerebrum. C) MRI showed postoperative manifestation of the sella region. There were the diploe signal disorder and thickening dura around the right frontal bone. An enhanced 1 cm-diameter patch was in the fundus of the 3rd encephalocoele. Pituitary and pituitary stalk were thin.

**Figure 3:**
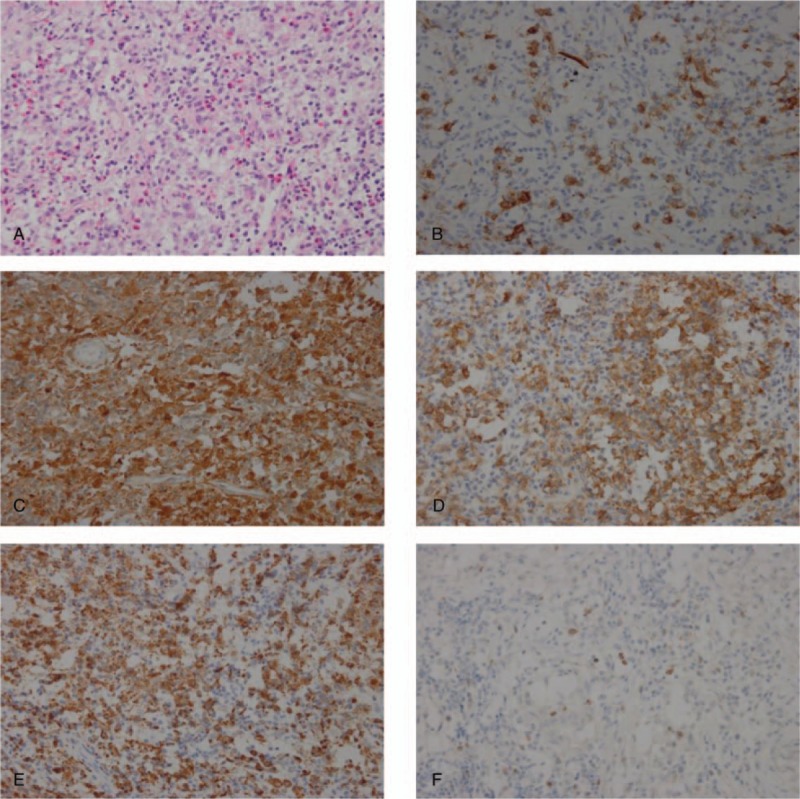
Photograph of surgical specimen. A) Scattered Langerhans cells were seen, with eosinophils and lymphocytes (HE staining, x400). And the lymphohistiocytic infiltration was positive to Langerin (B), S-100 protein (C) and to CD1a (D), CD68 (E), and Ki-67 (F) antigen markers (x400).

**Figure 4:**
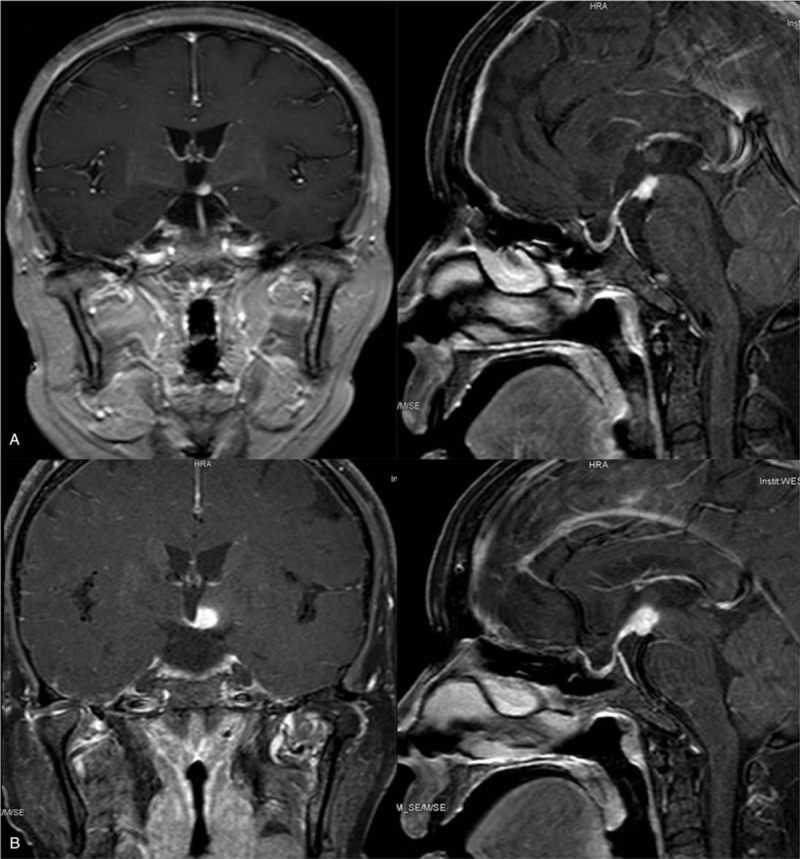
Enhanced MRI of sella region in 2016 (A) and 2017 (B). A) Enhanced MRI in 2016 showed bone flap of the right frontal bone with adjacent meninges thicking, and long T1 and T2 signal in bilateral centrum semiovale. Encephalocoele and cisterns showed no evident abnormalities. There was an enhanced nodule (0.8 cm) in the left hypothalamus. B) Enhanced MRI in 2017 showed enhanced nodule (1.4 cm) in the left hypothalamus and pituitary stalk thicking. Long T1 and T2 signal located in bilateral centrum semiovale. Encephalocoele and cisterns were normal.

The patient had improvement after radiosurgery and was discharged on the following day. However, 5 years later, the patient came to West China Hospital with weakness on June 2015. Hormone measurements revealed hypofunction of both hypophysial-thyroid axis and hypophysial-gonadal axis (Table 1B). Other laboratory profiles were shown in Table 1A. Enhanced head computed tomography (CT) showed lesions (1.6 × 1.3 cm) in the sella region, and showed homogeneous enhancement, but no bony lesions (Fig. 2A). Extra-lesions were neither found on Chest CT nor abdominal ultrasonography. Recurrence of sella region tumor was suspected based on the above findings, and tumorectomy was performed and levothyrocine was given. Histologic examination of the surgical specimen showed eosinophils and lymphocytes infiltration (Fig. 3A). And the lymphohistiocytic infiltration was positive to Langerin (Fig. 3B), S-100 protein (Fig. 3C) and to CD1a (Fig. 3D), CD68 (Fig. 3E), and Ki-67 (Fig. 3F) antigen markers. Thus, central nervous system (CNS) LCH (hypothalamic-hypophysial axis restricted) was diagnosed. Post-operation CT (Fig. 2B) and MRI (Fig. 2C) showed no evident abnormalities. She was on regular follow up in our outpatient service since discharged. One year later, the patient came to West China Hospital of Sichuan University again because of fatigue, and further hypofunction of hypophysial-adrenal axis was noted (Table 1B). And MRI of sella region on June 2016 showed enhanced nodular shadow (about 0.8 cm) on the left hypothalamus (Fig.4A). Though recurrence was considered, observation only was suggested due to the small size of the lesion. Levothyrocine together with prednisone was given. And in the latest MRI on May 2017, the nodular shadow became larger (about 1.4 cm) (Fig. 4B). Thus, chemotherapy (prednisone and anti-inflammatory drugs such as rofecoxib) was given. Active vitamin D and bisphosphonate therapy including zoledronate was used to treat her osteoporosis. At 12-month follow-up, no local reoccurrence was noticed.

## 3 Discussion

Langerhans’ cell histiocytosis, a group of diseases characterized by clonal proliferation of immature dendritic cells, mainly affects children. The accurate pathogensis of LCH is unknown, but there is a controversy over whether LCH is a clonal proliferative disease or an inflammatory disease.^[12,13]^ The incidence of LCH is 5 per million individuals according to Surveillance, Epidemiology, and End Results (SEER) Program database (https://seer.cancer.gov/). And the incidence of adult-onset LCH is estimated to be 1 to 2 per million populations per year.^[14]^ Any organ or system of human body, including bone, skin, and pituitary can be involved.^[15–19]^ And manifestations of LCH are totally diverse according to the sites and extents of the lesions. All lead to its high rate of misdiagnosis and missed diagnosis.

According to previous studies, the top 3 affected organs of LCH are bone (80% of patients), skin (33%), and pituitary (25%). Other less involved organs are liver (15%), lung (15%), lymph node (5–20%), central nervous system (CNS) LCH excluding pituitary (2–4%).^[15,20,21]^ Single systemic disease (SS-LCH) and multisystemic disease (MS-LCH) are two kinds of LCH based on the extent of involvement at diagnosis. Though isolated CNS LCH is reported, CNS LCH is mainly diagnosed as multisystemic disease, and the majority of CNS LCH patients are men.^[21,22]^ In this article, we present a rare case about a solitary hypothalamic-hypophysial axis involved female adult who develop central diabetes insipidus (CDI) and anterior pituitary hormone deficiency (APD).

Though difficult to be diagnosed, some manifestations can be meaningful when CNS is involved. The most common manifestations of CNS LCH are diabetes insipidus and neurodegeneration. And in 15% to 30% of these patients tend to develop CDI.^[15,20,23]^ In addition, these patients incline to develop APD.^[24,25]^ In a word, LCH should be highly suspected in patients who develop intracranial mass and CDI with or without APD.

The typical structure of the LCH affected tissues shows a mixture of immature dendritic cells and recruited inflammatory cells, including lymphocytes, eosinophils, and macrophages. Langerhans’ cells of the lesional region have abundant pink plasma, a coffee-bean like nucleus, and immunohistochemical positive staining for CD1a, S100, and (or) CD207 (Langerin).^[15,26–29]^ LCH diagnosis should always be founded on such positive immunohistochemical findings in addition to clinical and radiological characterisics. Therefore, multi-disciplinary team (MDT) treatment mode is necessary in such rare cases. In this case, endocrinologist recognized the possible CNS LCH on the basis of clinical and radiological characteristics and transferred the patient to neurosurgeon, then, neurosurgeon completed the surgery and obtained the lesional specimen, and the pathologist assisted to establish the final diagnosis of CNS LCH.

Till now, there is no defined standard therapeutic approach to adult. Depending on the extent of the clinical symptoms and signs, the lesional sizes and locations, and the variety of the clinical courses (self-limited or rapidly progressed), treatment approaches vary from observation only to systemic chemotherapy.^[15,20]^ According to current standard treatment in children, complete surgical excision achieves good efficacy only in treating isolated diabetes insipidus patients.^[15]^ But post-surgical recurrence is showed in some studies.^[30]^ Systemic treatment (vinblastine and steroid or 2-CdA monotherapy) is indicated when patients develop visual field alterations or an increase of lesional volume or brain (except pituitary stalk) or meningeal lesion is documented on the sequential MRI.^[15,31,32]^ The presented case showed these criterions were suitable for adults as well. Gamma knife radiosurgery was showed to be effective by one case in a 48 months’ follow-up.^[30]^ However, it should not be considered as first-line treatment approaches. What is more, hormonal replacements are recommended when APD occurs. With the cooperation of endocrinologist, neurosurgeons and pathologists, the patient had good clinical efficacy received treatment with multi-disciplinary team (MDT).

## 4 Conclusion

For LCH, though difficult to be diagnosed and none defined standard therapeutic approach to adults, some manifestations can be meaningful when central nervous system (CNS) is involved. Surgery should be considered if there are neurological symptoms or histological diagnosis. The present study showed that multi-disciplinary team (MDT) treatment is helpful for better clinical efficacy.

## Acknowledgments

The authors gratefully acknowledge the support of the nursing and technical staff of the Division of Endocrinology and Metabolism in West China Hospital for patient referrals and their excellent care of the patient with LCH. We would like to thank our patient and her daughter for all their help and enthusiasm. We would like to acknowledge Subrata Chakrabarti (Professor and Chair of Pathology and Laboratory Medicine, Western University, London, ON, Canada.) for help with the manuscript.

## Author contributions

**Conceptualization:** Huiwen Tan.

**Data curation:** Huiwen Tan, Kai Yu, Zhengmei An.

**Formal analysis:** Huiwen Tan, Yu Kai

**Investigation:** Huiwen Tan, Zhengmei An, Jianwei Li.

**Resources:** Huiwen Tan, Jianwei Li, Zhengmei An

**Supervision:** An Zhengmei, Yu Yerong.

**Validation:** Huiwen Tan, Jianwei Li, Yerong Yu

**Writing – original draft:** Huiwen Tan, Yu Kai.

**Writing – review & editing:** Yerong Yu, Zhengmei An, Jianwei Li.

Huiwen Tan orcid: 0000-0002-0451-8283.
